# How Well Is Ethiopia’s Moderate Acute Malnutrition Program Implemented?

**DOI:** 10.1016/j.cdnut.2026.109417

**Published:** 2026-06-26

**Authors:** Tarik Taye, Seifu Hagos Gebreyesus, Alinoor Mohamed Farah, Yakob Desalegn, Mark Spigt, Aweke Kebede, Beza Yilma, Robert Ackatia-Armah, Kemeria Barsenga

**Affiliations:** 1 Department of Family Medicine, School CAPHRI, Care and Public Health Research Institute, Maastricht University, Maastricht, The Netherlands; 2 Department of Nutrition and Dietetics, School of Public Health, College of Health Sciences, Addis Ababa University, Addis Ababa, Ethiopia; 3 Department of Community Medicine, General Practice Research Unit, University of Tromsø, Tromsø, Norway; 4 Nutrition Programme, World Food Programme (WFP), Addis Ababa, Ethiopia

**Keywords:** moderate acute malnutrition, implementation fidelity, community health, chart review, mid-upper arm circumference (MUAC)

## Abstract

**Background:**

The success of moderate acute malnutrition (MAM) management depends on adherence to protocol.

**Objectives:**

This study aimed to evaluate implementation fidelity of MAM management for children aged <5 y in Ethiopia through a retrospective chart review of 817 cases across 5 regions, representing diverse agroecologic zones.

**Methods:**

Data were collected from 101 health posts using systematic random sampling, and a structured checklist was used to extract data on sociodemographic status, protocol adherence, and follow-up care exposure. Data were managed digitally using Kobo Toolbox and analyzed with R version 20255.05.0, employing normality assessments, descriptive statistics, and nonparametric comparisons.

**Results:**

Admission protocols were well followed, with 95.7% of children admitted based on mid-upper arm circumference (MUAC) ≥11.5 cm and <12.5 cm. However, only 70.2% of children met the MUAC ≥12.5 cm discharge criteria, indicating weaker adherence at discharge. Follow-up care was limited; only 19% of children received deworming postadmission, and 38.6% of charts had incomplete deworming records. Most children (86.5%) attended the expected 3–8 visits, whereas 10.2% had >8 visits, predominantly in Afar and Sidama. Ready-to-use supplementary foods were the primary supplementary foods used in all regions. Regarding treatment duration, 84.4% of children remained in care for 3–16 wk, whereas 12.7% stayed beyond 16 wk, especially in Afar and Sidama. Target MUAC was achieved within 3–14 wk for 72.1% of children, but 22.6% required >14 wk, with prolonged recovery common in Afar (73.8% past 14 wk).

**Conclusions:**

Overall, admission fidelity was high, but gaps existed in discharge compliance, chart completeness, and follow-up care exposure. Regional disparities were pronounced in Afar and Sidama, where longer recovery times and higher visit frequencies were observed. Improving postadmission deworming and investigating regional differences are critical for enhancing MAM program effectiveness.

## Introduction

An estimated 45.4 million children aged <5 y suffer from acute malnutrition every year globally, contributing to nearly half of the overall mortality among children aged <5 y [1,2]. Africa bears the highest rate of acute malnutrition, accounting for 27.1% of the global burden [1]. Ethiopia is one of the hotspots for the acute malnutrition crisis in Africa, with an 11% national prevalence of acute malnutrition among children aged <5 y [3]. This makes acute malnutrition of high public health significance for the country, as per the WHO thresholds for the prevalence of acute malnutrition [4].

WHO recommends that countries implement a community-based nutrition screening program using trained community health workers (CHWs) for the assessment, classification, and management or referral of children with acute malnutrition [5]. Ethiopia has adopted WHO’s evidence-informed best practices and recommendations into its guidelines. Integration of the management of acute malnutrition with community health systems in Ethiopia was estimated to have saved 437,654 child deaths between 2008 and 2020 [6]. On the basis of the recommendations from the WHO and lessons from its National Nutrition Program, the Ministry of Health, Ethiopia, revised its national guidelines in May 2019 to integrate a Targeted Supplementary Feeding Program for the management of moderate acute malnutrition (MAM) among children aged 6–59 mo and ensure the continuum of care. This is an important transition because MAM affects a significantly larger number of children compared with severe acute malnutrition (SAM) and serves as a precursor to SAM [7], hence intensifying the reduction of the overall burden of acute malnutrition. In contrast, suboptimal management of MAM can lead to severe health complications, such as impaired immune function, increased susceptibility to infectious diseases, stunting, and impaired cognitive and physical development [8]. Therefore, ensuring fidelity of the guidelines for the management of MAM is not merely about managing the current nutritional status of the child, but rather preventing clinical complications, mitigating long-term developmental deficits, and ultimately contributing toward the National Health Sector Medium-term Development and Investment Plan and the global World Health Assembly nutrition targets [9,10].

Implementation fidelity refers to the extent to which programs are executed in accordance with the intended design by program developers [11]. Assessing fidelity shows whether an intervention works on its own and separates the intervention’s effects from errors in its delivery. In addition, understanding fidelity helps to repeat programmatic success in real-world settings. Without a good understanding of fidelity, conclusions about an intervention’s success will not be accurate [12]. However, only limited studies documented how well acute malnutrition management programs are implemented as per the protocol [13]. A retrospective cohort study that recently assessed time to recovery for children with MAM in Darolebu district, Eastern Ethiopia, showed that the recovery rate fell short of international standards, with a median recovery time of 16 wk [14]. This suggests the need for a thorough evaluation of fidelity of the MAM management to ensure optimal treatment outcomes. Because management of MAM was only recently integrated into the overall management of acute malnutrition in Ethiopia, little is known about the overall fidelity of the program. Hence, this study evaluated the implementation fidelity of MAM management protocols for children aged <5 y in selected regions of Ethiopia using facility chart review data. Specifically, the study used a modified conceptual framework for implementation fidelity from Carroll et al. [15] to measure adherence to standardized admission and discharge criteria for MAM, examine consistency of follow-up care practices, including medical treatment and follow-up frequency, and examine regional variations in protocol implementation of the MAM program.

## Methods

### Study setting and design

A facility-based retrospective chart review was conducted to assess implementation fidelity for the management of MAM among children aged <5 y. The Federal Democratic Republic of Ethiopia is divided into 12 regions and 2 city administrations, with 77% of the total population residing in rural areas [9,16]. The study covered 5 of the 12 regions, namely Afar, Oromia, Sidama, Somali, and Southern Nations, Nationalities, and Peoples’ (SNNP) Region, selected based on purposive sampling to represent the diverse agroecologic geographies in the country.

Primary health care (PHC) in Ethiopia is structured across primary hospitals, health centers, and satellite health posts. Health posts are the smallest PHC delivery units covering a population of 3000 to 5000 [17]. Data for this study were collected from health posts through retrospective chart reviews of children aged 6–59 mo enrolled for MAM care across the 5 regions.

### Sample size and sampling methods

The sample size was calculated using OpenEpi (version 3.01; developed by Dean AG, Sullivan KM, and Soe MM; Atlanta, Georgia, USA) open-source online calculator. The assumptions for sample size calculation included an anticipated 55.4% proportion of the overall adherence to standardized admission criteria for MAM as the primary outcome [14], a ±5% margin of error, and a confidence level of 95% with a design effect of 2.0. This resulted in a total sample size of 760 charts.

A multistage sampling methodology was used to select 1 woreda with a MAM program from each of the 5 regions. To ensure agroecological representation, the 5 woredas were further allocated across 3 agrarian and 2 pastoralist woredas. A proportional allocation method was used to set the number of health posts across each woreda based on their respective sizes. Data were collected between November 2024 and December 2024, and 101 health posts were included in the analysis (Table 1). Ten charts were randomly selected from children admitted for MAM care and treatment between September 2022 and July 2024. All the children enrolled were registered using standard patient charts for MAM management, and the charts were reviewed using a structured checklist. The checklist covered sociodemographic information of the child enrolled, nutritional status during admission to care, type of care provided during follow-up, type and quantity of supplementary food provided, and outcomes of the follow-up.

**TABLE 1:** Allocation of health posts across the 5 regions

	Regions					Total
	Afar	Oromia	Sidama	SNNP	Somali	
Number of health posts	11	31	18	26	15	101
Number of charts reviewed	110	246	171	139	151	817

Abbreviation: SNNP, Southern Nations, Nationalities, and Peoples.

### Data collection

The data were collected as part of a larger implementation research project on the integration of the management of MAM into the Ethiopian health system. We used a structured data extraction checklist, Supplemental Table 1, to carry out the chart review based on sociodemographic and care-related variables extracted from the MAM admission and follow-up register as per the national guideline. The checklist assessed variables across the 3 fidelity domains: adherence to admission and discharge protocols, exposure to follow-up care, and children’s responsiveness. For adherence to protocol, the variables included age of the child at admission, mid-upper arm circumference (MUAC) at admission, and MUAC at discharge. Variables such as deworming provision, number of visits after admission, type of supplementary food provided, and quantity of supplementary food provided per visit were collected to assess the child’s exposure to follow-up care. Finally, children’s responsiveness to care was assessed using the duration of stay in weeks and the week in which the target MUAC was achieved. To ensure high data accuracy and reliability, data collectors underwent rigorous 3-d training covering study objectives, chart selection protocol, and variable definition. The chart review was managed digitally using Kobo Toolbox (developed by the Harvard Humanitarian Initiative; Cambridge, Massachusetts, USA) for real-time data validation measures.

### Data management and analysis

**FIGURE 1:**
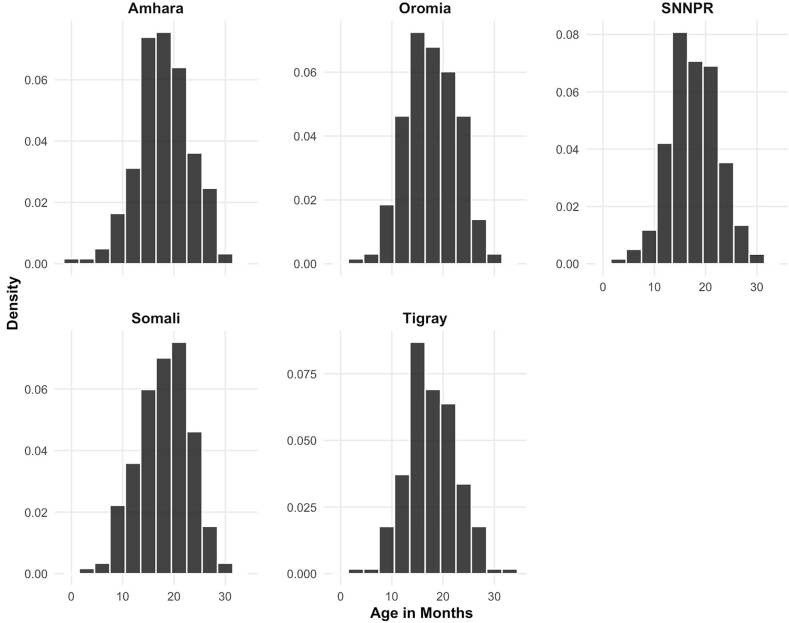
Age distribution across the 5 regions. SNNPR, Southern Nations, Nationalities, and Peoples’ Region.

**FIGURE 2:**
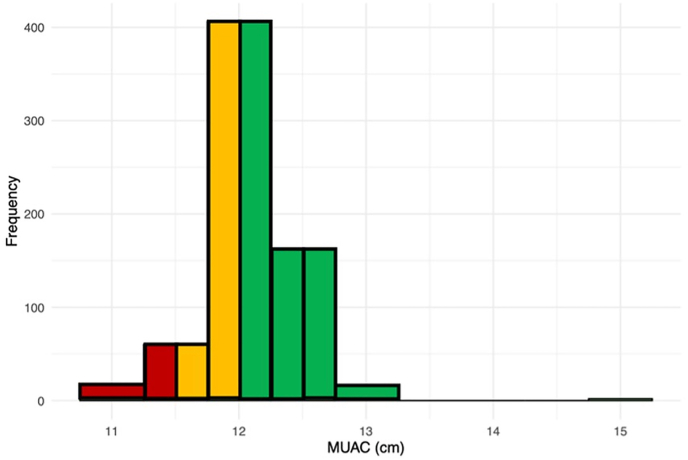
Distribution of mid-upper arm circumference (MUAC) at admission.

**FIGURE 3:**
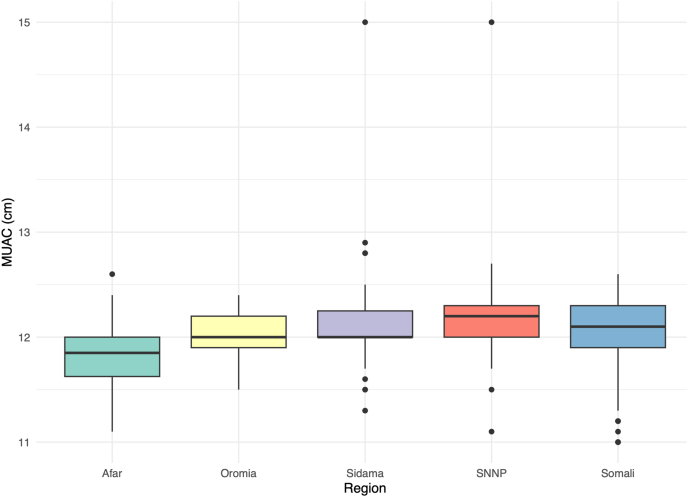
Mid-upper arm circumference (MUAC) at admission distribution by regions. SNNP, Southern Nations, Nationalities, and Peoples.

**FIGURE 4:**
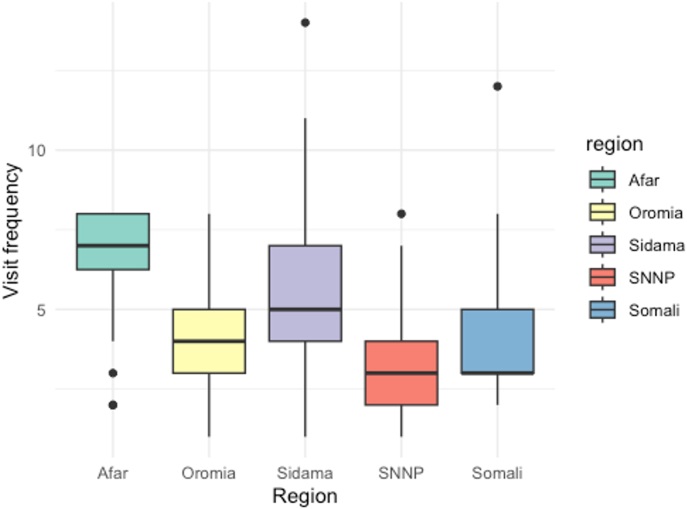
Visit frequency by region. SNNP, Southern Nations, Nationalities, and Peoples.

Data were exported to R (version 2025.05.0; Posit, Inc.; Boston, Massachusetts, USA) for cleaning and analysis, which involved addressing outliers, resolving inconsistencies, and managing missing data. Charts with only a few missing variables underwent a regression imputation process to preserve the statistical power of the study. For each variable containing missing values, a linear prediction model was constructed using complete cases across regional strata. For example, missing values for child’s age were imputed using a linear model predicted by baseline MUAC and number of visits. Similar separate predictive models were run sequentially for MUAC at admission, MUAC at discharge, number of visits, weeks of stay, week MUAC achieved, and number of sachets per visit. Predicted values were rounded to the nearest integer to maintain biological plausibility for further analysis. A comprehensive profile of missingness for these 7 variables before imputation is found in Supplemental Table 2. Descriptive statistics were assessed using frequency distributions. The appropriate measures of central tendency for adherence to the admission and discharge protocol, exposure to follow-up care, and children’s responsiveness to the management of MAM were used based on a modified implementation fidelity framework from Carroll et al. [15]. To compare fidelity across the 5 regions, continuous variables were first observed using histograms with a theoretical normal density curve and box plots to check for normality (FIGURE 1, FIGURE 2, FIGURE 3, FIGURE 4). The visual inspection indicated nonnormality, which was further assessed by testing all continuous variables for normality using the Shapiro test. All *P* values were <0.05, validating the visually observed nonnormality.

On the basis of the results of the normality tests, a nonparametric Kruskal–Wallis test was used to compare the median differences in adherence with admission and discharge protocols, exposure to follow-up care, and children’s responsiveness across the 5 regions. For variables with a significant difference on the Kruskal–Wallis test, a post hoc assessment was performed using Dunn’s test with Bonferroni correction to verify independent pairwise differences across the 5 regions. In addition, for the 2 categorical variables, namely, type of supplementary food provided and provision of deworming, a contingency table was developed to carry out a χ^2^ test of independence. The contingency table for the type of supplementary food provided had >20% of the cells with values of <5; hence, Fisher’s exact test was used for precision.

### Ethical consideration

Before data collection, ethical clearance was secured from the Research Ethical Committee of the School of Public Health and the Institutional Review Board (IRB) at the College of Health Sciences at Addis Ababa University with Protocol Approval Number 116/22/SPH on 2 January, 2023. Furthermore, permission was granted by the Federal Ministry of Health, respective regional health bureaus, and health facilities before data collection. Primary data extraction from health facility charts commenced on 23 November, 2024. Anonymity of the charts was maintained throughout data collection and analysis, ensuring data protection. Data were stored on a secure computer that could only be accessed by the investigators responsible for data verification and statistical modeling (TT, SHG, AMF, YD, MS, AK, BY, RA-A, and KB).

## Results

### Background characteristics

Table 2 describes background characteristics of the children whose charts were reviewed. Of the 817 charts reviewed, 175 (21.4%) were missing 1 or more data elements. The data incompleteness was more alarming in the Afar region, where half of the charts reviewed (48%) were missing 1 or more data elements, whereas Oromia had the lowest rate of missing data (7%). Further analysis was conducted to assess the extent of missing data elements, and charts with only a few missing elements underwent a regression imputation process to maintain the study power. After regression imputation, only 24 charts (2.9%) were found to be ineligible and excluded from the study. A total of 793 charts were included in the analysis.

**TABLE 2:** Descriptive statistics of children enrolled for MAM care by region

	Afar		Oromia		Sidama		SNNP		Somali		Total	
	*n*	%	*n*	%	*n*	%	*n*	%	*n*	%	*n*	%
Eligible charts (*N* = 817)	107	97.3	237	96.3	165	96.5	135	97.1	149	98.7	793	97.1
Age (mo)												
<6	3	2.8	0	0.0	0	0.0	2	1.5	0	0.0	5	0.6
6–23	70	65.4	144	60.8	78	47.3	80	59.3	45	30.2	417	52.6
24–59	34	31.8	91	38.4	85	51.5	52	38.5	103	69.1	365	46.0
>60	0	0.0	2	0.8	2	1.2	1	0.7	1	0.7	6	0.8
Total	107	100.0	237	100.0	165	100.0	135	100.0	149	100.0	793	100.0
MUAC at admission (cm)												
≤11.5	3	2.8	0	0.0	1	0.6	1	0.7	14	9.4	19	2.4
11.6–12.5	102	95.3	237	100.0	159	96.4	132	97.8	129	86.6	759	95.7
>12.5	2	1.9	0	0.0	5	3.0	2	1.5	6	4.0	15	1.9
Total	107	100.0	237	100.0	165	100.0	135	100.0	149	100.0	793	100.0
MUAC at discharge (cm)												
≤11.5	1	0.9	0	0.0	1	0.6	0	0.0	0	0.0	2	0.3
11.6–12.5	50	46.7	55	23.2	60	36.4	25	18.5	44	29.5	234	29.5
>12.5	56	52.3	182	76.8	104	63.0	110	81.5	105	70.5	557	70.2
Total	107	100.0	237	100.0	165	100.0	135	100.0	149	100.0	793	100.0
Frequency of visits												
1–2	3	2.8	1	0.4	5	3.0	17	12.6	0	0.0	26	3.3
3–8	70	65.4	232	97.9	121	73.3	117	86.7	146	98.0	686	86.5
>8	34	31.8	4	1.7	39	23.6	1	0.7	3	2.0	81	10.2
Total	107	100.0	237	100.0	165	100.0	135	100.0	149	100.0	793	100.0
Duration of stay (wk)												
<3	3	2.8	1	0.4	5	3.0	6	4.4	8	5.4	23	2.9
3–16	71	66.4	233	98.3	122	73.9	112	83.0	131	87.9	669	84.4
>16	33	30.8	3	1.3	38	23.0	17	12.6	10	6.7	101	12.7
Total	107	100.0	237	100.0	165	100.0	135	100.0	149	100.0	793	100.0
Week MUAC achieved												
<3	2	1.9	9	3.8	12	7.3	15	11.1	4	2.7	42	5.3
3–14	26	24.3	216	91.1	107	64.8	90	66.7	133	89.3	572	72.1
>14	79	73.8	12	5.1	46	27.9	30	22.2	12	8.1	179	22.6
Total	107	100.0	237	100.0	165	100.0	135	100.0	149	100.0	793	100.0
Type of supplementary food												
CSB++	0	0.0	1	0.4	0	0.0	3	2.2	1	0.7	5	0.6
RUSF	107	100.0	236	99.6	165	100.0	130	96.3	131	87.9	769	97.0
Not reported	0	0.0	0	0.0	0	0.0	2	1.5	17	11.4	19	2.4
Total	107	100.0	237	100.0	165	100.0	135	100.0	149	100.0	793	100.0
Amount of supplementary food provided per visit												
<7 sachets	7	6.5	1	0.4	1	0.6	1	0.7	2	1.3	12	1.5
7–15 sachets	0	0.0	1	0.4	0	0.0	0	0.0	0	0.0	1	0.1
16–30 sachets	94	87.9	229	96.6	150	90.9	99	73.3	133	89.3	705	88.9
>30 sachets	6	5.6	6	2.5	14	8.5	35	25.9	14	9.4	75	9.5
Total	107	100.0	237	100.0	165	100.0	135	100.0	149	100.0	793	100.0
Deworming												
Yes	40	37.4	18	7.6	37	22.4	14	10.4	42	28.2	151	19.0
No	23	21.5	124	52.3	23	13.9	63	46.7	73	49.0	306	38.6
Not reported	44	41.1	95	40.1	105	63.6	58	43.0	34	22.8	336	42.4
Total	107	100.0	237	100.0	165	100.0	135	100.0	149	100.0	793	100.0

Abbreviations: CSB, corn-soya blend; MAM, moderate acute malnutrition; MUAC, mid-upper arm circumference; RUSF, ready-to-use supplementary foods; SNNP, Southern Nations, Nationalities, and Peoples.

### Adherence to protocol

Most of the children admitted to MAM care were between 6 mo and 5 y, which is the age group the program is designed to serve. Only 1.4% of the children admitted were outside this age range. Almost all of the children enrolled for MAM management (95.7%) had an admission MUAC between 11.6 and 12.5 cm, consistent with the admission protocol. However, few children (2.4%) were severely malnourished and should have been enrolled in SAM management, and another 1.9% had a normal MUAC during admission. Of the 19 severely malnourished children who were mistakenly admitted to MAM care, 14 were from the Somali region. Moreover, the Sidama and Somali regions contributed to two-thirds of the children admitted with a normal MUAC.

Similarly, MUAC at discharge showed a good adherence to protocol, with 7 of 10 children discharged after having a MUAC of >12.5 cm, which qualifies as a successful treatment outcome. However, one-third of the children enrolled were discharged with a MUAC between 11.6 cm and 12.5 cm, inconsistent with the protocol and necessitating readmission to care to prevent adverse clinical outcomes or further progression to SAM; Afar and Sidama each contributed the highest proportions of these children at 46.7% and 36.4%, respectively.

### Exposure to follow-up care

Of the 793 charts analyzed, alarmingly, only 19% of the children received deworming after admission to MAM care; in addition, a significant number of charts did not document whether children received deworming (38.6%), leading to poor treatment outcomes. Between admission and discharge, most children (86.5%) had 3–8 visits, which is consistent with the expected visit frequency. Close to 10.2% of the children admitted to care had >8 visits on record, highlighting a prolonged duration of treatment or more frequent visits than recommended. Afar and Sidama are the 2 regions with the most frequent visits by children during care. The type of supplementary foods provided is also consistent with the recommendation from the national guideline, as ready-to-use supplementary foods (RUSF) were the most widely used supplementary food across the 5 regions, except for 5 children from 3 different regions who received corn-soya blend (CSB) ++. After admission to MAM care, caretakers are expected to receive ≥15 sachets of supplementary foods enough to feed the child until the next follow-up. On the basis of the results, the majority of the children (88.9%) received 16–30 sachets of supplementary foods per visit. The SNNP region showed a significantly higher proportion of children (25.9%) receiving 30 sachets or more per visit, whereas the Afar region had a few children (6.5%) who were provided with ≤7 sachets of supplementary foods per visit.

### Children’s responsiveness to care

Most of the children admitted to MAM care (84.4%) stayed in care for 3–16 wk. This is consistent with the expected duration for MAM treatment. Approximately 12.7% of the children had an extended stay of >16 wk, indicating poor responsiveness to care. The Afar and Sidama regions were the major contributors to prolonged stay in care.

The majority of the children admitted for MAM treatment (72.1%) achieved their target MUAC between 3 and 14 wk. However, it took over 14 wk to achieve the target MUAC for 22.6% of children. Afar region showed the most prolonged duration to achieve the target MUAC, with 73.8% achieving their target MUAC 14 wk after admission.

### Comparison of fidelity across the 5 regions

The Kruskal–Wallis test showed that all continuous variables related to protocol adherence and follow-up care exposure differed significantly across the 5 regions (Table 3).

**TABLE 3:** Kruskal–Wallis test result

Kruskal–Wallis rank sum test	Statistic	*df*	*P* value∗
Age (mo)	67.432	4	<0.001
MUAC at admission	75.79	4	<0.001
MUAC at discharge	47.164	4	<0.001
Number of visits	202.5	4	<0.001
Weeks of stay	122.24	4	<0.001
Week MUAC achieved	156.24	4	<0.001
Number of sachets per visit	196.85	4	<0.001

Abbreviation: MUAC, mid-upper arm circumference.

∗ All *P* values showed statistical significance.

On the basis of the results of the Kruskal–Wallis test, a post hoc Dunn’s test with Bonferroni correction was performed for pairwise comparison of the differences across the 5 regions (Table 4).

**TABLE 4:** Results of the pairwise Dunn's test with a Bonferroni correction

Comparison	Age (mo)		MUAC at admission		MUAC at discharge		Number of visits		Weeks of stay		Week MUAC reached		Packets per visit	
	*Z*	*P* value	*Z*	*P* value	*Z*	*P* value	*Z*	*P* value	*Z*	*P* value	*Z*	*P* value	*Z*	*P* value
Afar–Oromia	–1.29	0.9847	–0.4	1	–3.76	<0.001∗∗∗	8.99	<0.001∗∗∗	9.24	<0.001∗∗∗	11.57	<0.001∗∗∗	–0.27	1
Afar–Sidama	–4.45	<0.001∗∗∗	–1.31	0.94298	–1.42	0.77166	3.08	0.0104∗	3.32	0.00445∗	6.71	<0.001∗∗∗	–0.28	1
Oromia–Sidama	–3.96	<0.001∗∗∗	–1.16	1	2.58	0.05006	–6.55	<0.001∗∗∗	–6.55	<0.001∗∗∗	–5.07	<0.001∗∗∗	–0.43	1
Afar–SNNP	–1.57	0.5813	–0.63	1	–6.17	<0.001∗∗∗	12.3	<0.001∗∗∗	8.42	<0.001∗∗∗	9.9	<0.001∗∗∗	–10.36	<0.001∗∗∗
Oromia–SNNP	–0.49	1	–0.33	1	–3.34	0.00416∗	5.05	<0.001∗∗∗	0.13	1	–0.61	1	–12.52	<0.001∗∗∗
Sidama–SNNP	3.01	0.0133	0.7	1	–5.36	<0.001∗∗∗	10.42	<0.001∗∗∗	5.84	<0.001∗∗∗	3.87	0.001∗∗∗	–11.26	<0.001∗∗∗
Afar–Somali	–6.6	<0.001∗∗∗	1.7	0.44492	–2.44	0.07371	8.26	<0.001∗∗∗	4.58	<0.001∗∗∗	9.42	<0.001∗∗∗	–4.12	<0.001∗∗∗
Oromia–Somali	–6.56	<0.001∗∗∗	2.5	0.0618	1.23	1	0	1	–4.74	<0.001∗∗∗	–1.47	0.70458	–5.09	<0.001∗∗∗
Sidama–Somali	–2.31	0.0595	3.35	0.00403∗∗	–1.17	1	5.88	<0.001∗∗∗	1.49	0.68702	3.19	0.00711∗∗	–4.33	<0.001∗∗∗
SNNP–Somali	–5.33	<0.001∗∗∗	2.56	0.0516	4.12	<0.001∗∗∗	–4.59	<0.001∗∗∗	–4.29	<0.001∗∗∗	–0.74	1	6.88	<0.001∗∗∗

Abbreviations: MUAC, mid-upper arm circumference; SNNP, Southern Nations, Nationalities, and Peoples.

Significance levels: ∗∗∗*P*<0.001, ∗∗*P*<0.01, ∗*P*<0.05.

According to the pairwise comparison, the number of follow-up visits and duration of stay were the 2 variables with the highest statistically significant variation across regions, with the exception of Afar and Sidama regions. Meanwhile, MUAC at admission showed the least significant difference across regions. Moreover, Afar and SNNP showed persistent and elevated variations across the different variables compared with the other 3 regions.

Similarly, regional comparison of the 2 categorical variables using the χ^2^ test and Fisher’s exact test revealed a statistically significant difference in the type of supplementary food provided and deworming across the 5 regions (Table 5).

**TABLE 5:** Results of the χ^2^ and Fisher’s exact test

Test	Test statistic	*df*	*P* value	Significance
χ^2^ test for deworming status by region	131.6	8	<0.001	∗∗∗
Fisher’s exact test for type of supplementary food provided by region	—	—	<0.001	∗∗∗

∗∗∗ Significant association.

The results showed that the Oromia region accounted for the least proportion of children with MAM dewormed (52.3%), whereas the Sidama region had the highest nonreported status of deworming (63.6%) after enrollment.

## Discussion

The study aimed at evaluating the implementation fidelity of MAM management for children aged <5 y across 5 regions of Ethiopia using facility chart review data. Fidelity was assessed based on adherence to protocol, exposure to follow-up care, and children’s responsiveness. Although most children admitted to MAM care were within the target age range and met admission MUAC criteria, discharge practices showed partial fidelity, with one-third of the children released having suboptimal MUAC, raising concerns of relapse. Follow-up care was uneven, with only 19% of children receiving documented deworming despite most children meeting the expected visit frequency and type of supplementary foods for treatment. Statistical comparisons confirmed significant regional disparities in follow-up frequency, duration of care, and deworming coverage, highlighting inconsistencies that may undermine optimal MAM management outcomes.

The review showed that 1 in 5 charts had 1 or more missing data elements. The findings differ from results of a systematic review that assessed the Health Management Information System and nutrition data in Ethiopia, which highlighted a 21% completeness of data at 90% level [18]. This could be attributed to the recent training of care providers and follow-up as part of the programmatic support after the national integration of MAM treatment.

With strong compliance with the admission criteria for MAM, the study showed that adherence to protocol is encouraging across the 5 regions, with some exceptions in the Somali region, where few children with SAM were enrolled in the MAM program. The admission criteria compliance rates were relatively higher than a similar study in Nigeria, where CHWs showed only 45% adherence. The sample size could have been a factor for the variation, with the Nigeria study having a smaller sample size. Moreover, the same study highlighted that the experience of CHWs was a positive predictor of high adherence to the MUAC cutoff [19]. A recent validation study that tested MUAC cutoff points vis-a-vis weight-for-age measures for diagnosis of MAM indicated that the optimal MUAC cutoff to diagnose MAM among children in the Somali region is 13.75 cm, indicating the 12.5 cm cutoff as a conservative estimate denoting the possibility of children with a normal MUAC to be admitted for MAM care [20]. In contrast, few children with SAM are admitted to MAM care in the Somali region. Furthermore, MUAC at discharge had relatively lower adherence to protocol across the 5 regions, with only 2 of 3 children meeting the MUAC for discharge criteria. Afar and Somali regions, in particular, showed higher rates of children discharged with a MUAC between 11.6 and 12.5 cm (inclusive). This could be due to the widespread challenges of poor documentation and record-keeping in pastoralist regions, coupled with the operational complexities faced with high caseloads, staff shortages, and insufficient programmatic supervision [21].

Exposure to care is an area where the highest regional variation was observed. Although most children enrolled in MAM care had adequate frequency of visits, only 1 of 5 children admitted were dewormed. In addition, 1 of 3 charts had no documentation of the history of deworming on record. This was particularly evident in the Sidama region, where 2 of the 3 charts had no record of deworming status, whereas half of the children enrolled in the Oromia and Somali regions did not receive deworming treatment. A retrospective facility-based chart review from Dire Dawa, Ethiopia, showed similar results, with only 25.1% of children receiving deworming after enrollment for the management of acute malnutrition, indicating similar challenges of poor compliance with deworming across urban centers [22]. Irregular supply and stock-outs were constantly cited in other studies as the main cause of poor deworming coverage during the management of acute malnutrition [23]. The type of nutritional supplementation provided for children with MAM was invariably RUSF, except for a few children who received CSB++. This is in line with the national guidelines for the management of acute malnutrition [24].

More than 8 of 10 children stayed under MAM care for 3–16 wk, consistent with the expected duration of stay as per the national guidelines. Dunn’s test with Bonferroni correction highlighted that the median duration of stay and frequency of follow-up visits were the 2 variables with the highest differences across regions. Afar region had the highest median duration of stay, leading to a higher median number of visits reported compared with other regions. In addition, Afar region was also found to have the longest median duration to achieve target MUAC, whereas Oromia and Somali regions showed a median duration of stay for target MUAC achievement that is most aligned with the expected 3–14 wk duration as per the national guideline for the management of acute malnutrition. The longest median duration recorded in Afar could be due to factors ranging from geographical and infrastructural challenges to weak health system capacity and socioeconomic and cultural constraints in the pastoralist community [25].

Overall, the regional variation observed across adherence to protocol, exposure to follow-up, and children’s responsiveness to care is driven by a complex interplay of structural and systemic barriers across the different regions. In pastoralist areas, the high rate of missing data and delayed recovery times stem from widespread challenges of poor documentation and record-keeping, compounded by operational complexities like heavy caseloads, acute staff shortages, and insufficient programmatic supervision [21]. Furthermore, these bottlenecks are exacerbated by broader geographical and infrastructural hurdles, weak localized health system capacity, and unique socioeconomic and cultural constraints inherent to pastoralist communities. These factors create a compounding disadvantage that impairs protocol adherence, strains follow-up care, and ultimately drives the start disparities in treatment timelines and programmatic fidelity.

### Strengths and limitations of the study

The study is representative of both agrarian and pastoralist regions of the country, providing insights into the management of MAM across multiple agroecological settings of rural Ethiopia. Moreover, the charts reviewed were from the year preceding data collection, providing fresh insights into the management of MAM in the 5 regions. The study is subject to limitations from the regression imputation carried out to address missing values from charts with incomplete data elements, which may have led to an underestimation of the magnitude of variability across the study regions. Finally, the study did not evaluate breastfeeding patterns or maternal lactation support during care, which represents a notable limitation given the foundational role of exclusive and continued breastfeeding in the management of MAM. Retrospective chart reviews often lack detailed lactation records, a persistent gap as highlighted in recent MAM literature [26].

In conclusion, the study evaluated the implementation of MAM management protocols across 5 regions of Ethiopia. Fidelity was measured through adherence to protocol, exposure to follow-up care, and children’s responsiveness to care. Although overall adherence to protocol was consistently high across all regions, significant variations were observed in chart incompleteness, exposure to follow-up care, and children’s responsiveness. These variations were particularly pronounced in the Afar and Sidama regions, highlighting the need for targeted interventions that channel resources, specialized staff training, and infrastructure support to the lowest-performing regions. Further research is also required to isolate specific drivers for the high variation in fidelity among the Afar and Sidama regions to support targeted interventions. A major gap was also identified in deworming children after admission, with only 1 of 5 children dewormed, indicating suboptimal follow-up care across regions. Finally, future research should integrate standardized child development indicators alongside routine anthropometric measurements to comprehensively evaluate the impact of MAM management and children’s response to care.

## Author contributions

The authors' responsibilities were as follows — TT, SHG, AMF, MS, AK, BY, YD, RA-A, KB: conceived and designed the study; TT, SHG, MS: performed the analysis; TT: drafted the manuscript; and all authors: provided critical conceptual revisions, read and approved the final manuscript.

## Data availability

Data described in the manuscript, code book, and analytic code will be made available upon request. All data underlying the findings are fully available.

## Ethics approval and consent to participate

The ethical considerations involved in this study were carefully observed to protect the rights, welfare, and privacy of the participants and in compliance with national and international ethical standards at all stages of the study. Furthermore, administrative permission was also granted from the Federal Ministry of Health, respective regional health bureaus, and health facilities before data collection.

## Funding

The authors reported no funding received for this study.

## Declaration of generative AI and AI-assisted technologies in the writing process

The authors declare that no generative AI or AI-assisted technologies were used in the writing of this manuscript.

## Conflict of interest

The authors report no conflicts of interest.
