# Supplementary material for: How Well Is Ethiopia’s Moderate Acute Malnutrition Program Implemented?
Source: Curr Dev Nutr. 2026 Jun 26;10(8):109417. doi: 10.1016/j.cdnut.2026.109417 (PMC13416640; doi:10.1016/j.cdnut.2026.109417)
Supplement: multimedia component 1 [file mmc1.docx]

**How Well Is Ethiopia’s Moderate Acute Malnutrition Program Implemented?**

First author: Tarik Taye

Supplementary Table 1: Missing values prior to imputation

| Region | Total Charts (N) | Age | | MUAC^1^ at Admission | | MUAC at Discharge | | Number of Visits | | Weeks of stay | | Week MUAC achieved | | Number of Sachets per visit | |
| --- | --- | --- | --- | --- | --- | --- | --- | --- | --- | --- | --- | --- | --- | --- | --- |
|  |  | n | % | n | % | n | % | n | % | n | % | n | % | n | % |
| Afar | 110 | 12 | 10.9 | 12 | 10.9 | 6 | 5.5 | 0 | 0 | 0 | 0 | 4 | 3.6 | 0 | 0 |
| Oromia | 246 | 7 | 2.8 | 5 | 2 | 5 | 2 | 5 | 2 | 6 | 2.4 | 5 | 2 | 5 | 2 |
| Sidama | 171 | 16 | 9.4 | 6 | 3.5 | 6 | 3.5 | 6 | 3.5 | 6 | 3.5 | 6 | 3.5 | 6 | 3.5 |
| SNNP^2^ | 139 | 2 | 1.4 | 4 | 2.9 | 5 | 3.6 | 2 | 1.4 | 3 | 2.2 | 5 | 3.6 | 2 | 1.4 |
| Somali | 151 | 22 | 14.6 | 10 | 6.6 | 20 | 13.2 | 9 | 6 | 1 | 0.7 | 20 | 13.2 | 5 | 3.3 |
| Total | 817 | 59 | 0.1 | 37 | 0.0 | 42 | 0.1 | 22 | 0.0 | 16 | 0.0 | 40 | 0.0 | 18 | 0.0 |

^1^Mid Upper Arm Circumference (MUAC)

^2^Southern Nations Nationalities and Peoples’ (SNNP)
