# Supplementary material for: How Well Is Ethiopia’s Moderate Acute Malnutrition Program Implemented?
Source: Curr Dev Nutr. 2026 Jun 26;10(8):109417. doi: 10.1016/j.cdnut.2026.109417 (PMC13416640; doi:10.1016/j.cdnut.2026.109417)
Supplement: multimedia component 2 [file mmc2.docx]

**How Well Is Ethiopia’s Moderate Acute Malnutrition Program Implemented?**

First author: Tarik Taye

Supplementary Table 2: Data extraction tool Missing values prior to imputation

| **Section 2a – U5 children chart sampled** |  |
| --- | --- |
| **(C_ID) Sampled Child ID** | Data set ID |
| **(201a) Date of admission** | DD/MM/YY |
| **(202a) Number of visits** | Number input |
| **(203a) Age of the child in MONTHS** | Number input |
| **(204a) MUAC^1^ at admission (cm)** | Number input (__.__) |
| **(205a) MUAC at discharge (cm)** | Number input (__.__) |
| **(206a) Week in which MUAC reached ≥12.5 cm** | Number input (__) |
| **(207a) Weight at admission (kg)** | Number input (__._) |
| **(208a) Weight at discharge (kg)** | Number input (__._) |
| **(209a) Length of stay in WEEKS** | Number input (__) |
| **(210a) Type of specialized nutritious food** | (01) RUSF or Plumpy sup (02) CSB++ rations (03) Not filled |
| **(211a) Number of sachets/packets provided per VISIT** | Number input |
| **(212a) Recived Deworming (Mebendazole/Albendazole)?** | (01) Yes (02) No (03) Not filled |
| **(213a) Action Indicated ?** | (01) Yes (02) No (03) Not filled |
| **(214a) If action indicated, is it Partial or Completed** | (01) Partial (02) Completed (03) Not filled |

^1^Mid Upper Arm Circumference
